# Small-Angle X-ray Scattering Analysis on the Estimation of Interaction Parameter of Poly(*n*-butyl acrylate)-*b*-poly(methyl methacrylate)

**DOI:** 10.3390/polym14245567

**Published:** 2022-12-19

**Authors:** Sang-In Lee, Min-Guk Seo, June Huh, Hyun-jong Paik

**Affiliations:** 1 Department of Polymer Science and Engineering, Pusan National University, Busan 46241, Republic of Korea; 2 LX MMA R&D Center, 188, Munji-ro, Yuseong-gu, Daejeon 34122, Republic of Korea; 3 Department of Chemical and Biological Engineering, Korea University, Seoul 02841, Republic of Korea; 4 Department of Life Sciences, Korea University, Seoul 02841, Republic of Korea

**Keywords:** acrylic block copolymer, Flory–Huggins interaction parameter, thermoplastic elastomer, small-angle X-ray scattering

## Abstract

The temperature dependence of the Flory–Huggins interaction parameter χ for poly(*n*-butyl acrylate)-*b*-poly(methyl methacrylate) (PBA-*b*-PMMA) was quantified from small-angle X-ray scattering (SAXS) analysis using random phase approximation (RPA) theory. It was found from the χ estimation (χ=0.0103+14.76/T) that the enthalpic contribution, $\chi_{H}$, a measure for temperature susceptibility of χ, is 1.7–4.5 folds smaller for PBA-*b*-PMMA than for the conventional styrene-diene-based block copolymers, which have been widely used for thermoplastic elastomers. This finding suggests that these fully acrylic components can be a desirable chemical pair for constituting terpolymers applied for thermally stable and mechanically resilient elastomers.

## 1. Introduction

The development of technologies of ligated anionic polymerization (LAP) and controlled radical polymerization (CRP) have allowed the commercialization of novel block copolymers with well-defined molecular architectures [1,2,3,4]. Various block copolymers are being produced using LAP and nitroxide-mediated polymerization (NMP) technology, the representative example being Kurarity by Kuraray and Nanostrength by Arkema. In particular, fully acrylic block copolymers [2,5,6,7,8,9,10], comprised of a rubbery poly(alkyl acrylate) block and glassy poly(alkyl methacrylate) block, have attracted steady interest in the thermoplastic elastomer (TPE) community because of the much higher weather resistance of poly(meth)acrylates when compared to traditional diene-based TPEs [11,12,13]. Well-designed triblock copolymers consisting of two terminal blocks with glassy segments and a middle block with rubbery segments can form glassy nanodomains embedded in a rubbery matrix, where chains bridging between two different glassy domains, which can efficiently persist the mechanical deformation, play a critical role for the overall mechanical properties of TPE. In this respect, the interaction between block components, usually quantified by the Flory–Huggins interaction parameter χ, is of critical importance in controlling the conformational and morphological behavior, which determines the mechanical performance of TPE.

Among many possible acrylic components, the block copolymers comprised of an *n*-butyl acrylate (BA) block and a methyl methacrylate (MMA) block are one of the promising species for TPE owing to their excellent properties, such as light sensitivity and oxidation stability. Previously, several studies have been carried out to synthesize PBA-PMMA copolymers with various chain architectures [2,5,6,7,8,9,10,14,15,16], some of which have reported their methods for the generation of elastomers and thermomechanical properties. Nonetheless, no works have been conducted for the temperature dependence of the interaction parameter χ(T) between PBA and PMMA despite its importance for thermomechanical properties, which are closely related to conformational and morphological behavior via χ(T). For instance, it has been theoretically reported that the fraction of bridging conformations between nanodomains, which contributes favorably to the mechanical performance of TPE, is proportional to $\chi^{-1/9}$[17]. This prediction suggests that introducing a constituent block pair with small χ with weak temperature dependence is advantageous for developing a TPE with enhanced thermal stability.

In this paper, we report the temperature dependence of the Flory–Huggins interaction parameter χ(T) between the two acrylic monomer species of BA and MMA by small-angle X-ray scattering (SAXS) measurements of a molten PBA-*b*-PMMA block copolymer using a random phase approximation (RPA) analysis. To do this, we synthesized the PBA-*b*-PMMA diblock copolymer with a carefully chosen molecular weight by ATRP, which allowed measuring the order-disorder transition (ODT) temperature within the investigated temperature range.

## 2. Materials and Methods

### 2.1. Materials

Butyl acrylate (TCI, 99%), methyl methacrylate (DAEJUNG CHEMICALS & METALS, Siheung, Korea 99.5%), and styrene (Junsei, Tokyo, Japan, 99.5%) were purified by passing through a basic alumina column before use. Copper(I) bromide (CuBr, Sigma-Aldrich, Seoul, Korea, 98.0%) and copper(I) chloride (CuCl, Sigma-Aldrich, 98.0%) were purified by stirring with glacial acetic acid, followed by filtering and washing the resulting solid four times with ethanol. The solid was dried under a vacuum for two days. Anisole (DAEJUNG CHEMICALS & METALS, 98.0%), (1-Bromoethyl)benzene (Sigma-Aldrich, 98%), copper(II) bromide (CuBr2, Sigma-Aldrich, 98.0%), copper(II) chloride (CuCl2, Sigma-Aldrich, 98.0%) and N,N,N${}^{\prime }$,N${}^{\prime \prime }$,N${}^{\prime \prime }$-pentamethyldiethylenetriamine (PMDETA, Sigma-Aldrich, 98%), and all other chemicals, internal standards, and solvents were used as received.

### 2.2. ATRP Synthesis

#### 2.2.1. Synthesis of Poly(n-butyl acrylate) (PBA) Macro Initiator

A dried 100 mL Schlenk flask was charged with CuBr (152.97 mg, 1.07 mmol), CuBr${}_{2}$ (4.86 mg, 0.02 mmol). Then, degassed butyl acrylate (30.0 mL, 208.27 mmol), (1-Bromoethyl) benzene (0.15 mL, 1.09 mmol), PMDETA (0.23 mL, 1.09 mmol) and anisole (20.0 mL) were added to the flask via N${}_{2}$ purged syringes. The mixture was reacted in an oil bath at 90 °C. At 40% monomer conversion (measured by gas chromatography), the polymerization was quenched by THF and exposed to air. Then, the reaction mixture was passed through a neutral alumina column to remove the Cu catalyst. After being concentrated using a rotary evaporator, the solution was dropped into methanol/water (8:2) to obtain the polymer as a precipitate. The molecular weight of the resulting polymer in SEC was determined as $M_{n}$ = 12,460 g/mol and $M_{w}/M_{n}$ = 1.084, where $M_{n}$ and $M_{w}$ are the number-averaged and the weight-averaged molecular weight, respectively.

#### 2.2.2. Synthesis of Poly(*n*-butyl acrylate)-*b*-p,oly(methyl methacrylate) (PBA-*b*-PMMA) Diblock Copolymer

A dried 50 mL Schlenk flask was charged with CuCl (122.0 mg, 0.91 mmol) and CuCl${}_{2}$ (7.50 mg, 0.02 mmol). After being sealed with a glass stopper, the flask was evacuated and back-filled with N${}_{2}$ three times. Then, degassed methyl methacrylate and PBA Macro initiator (1 g, 0.09 mmol) resolved in anisole (10.0 mL) were added to the flask via N${}_{2}$ purged syringes. The mixture was reacted in an oil bath at 85 °C. The polymerizations were quenched by THF and exposed to air. Then, the reaction mixtures were passed through a neutral alumina column to remove the Cu catalyst. After being concentrated using a rotary evaporator, the solutions were dropped into methanol to obtain the polymer as precipitates. After filtration and drying under a vacuum, the polymers were isolated as white powder. The molecular weight of the resulting polymer in SEC was determined as $M_{n}$ = 23,470 g/mol and $M_{w}/M_{n}$ = 1.151.

#### 2.2.3. Conversion Analysis

In all the ATRP stages, monomer conversion was confirmed by HP 5890 gas chromatography (GC) equipped with an HP101 column. The number-averaged molecular weight and the dispersity were determined by SEC calibrated with PMMA standards.

### 2.3. SAXS Measurements

SAXS measurements were performed at the 4C SAXS II beamline of the Pohang Light Source II (PLS II) with 3 GeV power at the POSTECH. A sample-to-detector distance of 3.00 m was used for SAXS. SAXS profiles were measured in situ at each temperature with the SAXS apparatus described elsewhere. The sample was placed in the sample chamber filled with nitrogen gas, and the temperature was controlled with an accuracy of +/−0.03 ${}^{\circ }$C. Since SAXS measurements were conducted in the temperature range of 170–230 ${}^{\circ }$C, precise temperature control was required for block copolymer samples to avoid the possible thermal degradation of PMMA [18,19]. GPC found no mass loss after SAXS measurements of samples. The profiles were desmeared for slit-height and slit-width effects and corrected for absorption, air scattering, and thermal diffuse scattering as described elsewhere [20]. The SAXS measurements were conducted during a heating process that started at 130 ${}^{\circ }$C. During the heating run, the PBA-*b*-PMMA sample was maintained for 1 h at a specific temperature to obtain thermal equilibrium as much as possible.

## 3. Results

**Figure 1:**
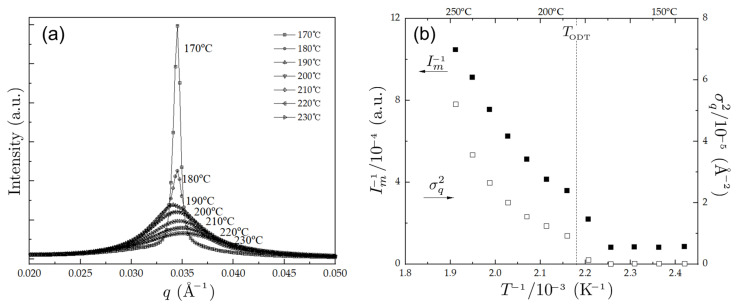
(**a**) SAXS profiles at various temperatures and (**b**) the inverse of the maximum scattering intensity ($I_{m}^{-1}$) and the half-width at half-maximum ($\sigma_{q}^{2}$) versus the inverse of the temperature for PBA-*b*-PMMA. The dashed line in (**b**) indicates the temperature at ODT, $T_{\mathrm{ODT}}$.

Figure 1a presents the SAXS profiles of PBA-*b*-PMMA obtained at various temperatures covering both disordered and ordered regimes. As seen in Figure 1, the broad SAXS curves in the high-temperature regimes become sharply peaked below *T* = 180 ${}^{\circ }$C, at which point the PBA-*b*-PMMA undergoes the order-disorder transition. This is manifested more clearly in Figure 1b, where the inverse of the maximum scattering intensity ($I_{m}^{-1}$ ) and the half-width at half-maximum ($\sigma_{q}^{2}$ ) are plotted against the reciprocal temperature ($T^{-1}$ ), where a jump-like discontinuity of $I_{m}^{-1}$ and $\sigma_{q}^{2}$ at 180 ${}^{\circ }$C can be seen.

Having identified the disordered and the ordered regime by locating the temperature at ODT ($T_{\mathrm{ODT}}$), the temperature-dependence of χ parameters between PBA and PMMA was estimated by RPA analysis at $T>T_{\mathrm{MF}}$, where $T>T_{\mathrm{MF}}$ is the crossover temperature from a mean-field disordered regime to the non-mean-field regime [21,22,23]. The RPA states that the scattered intensity I(q) at scattering vector *q* in the disordered state is linearly proportional to ${\left[\Gamma \left(q,R_{1},R_{2}\right)-2\chi \right]}^{-1}$ where Γ is referred to as the second vertex function related to the single chain density correlation functions in the ideal state, and $R_{\alpha }$ is the root-mean-square radius of gyration of the component (α = 1: PBA, α = 2: PMMA). The estimation of χ at each temperature was then obtained by fitting $I\left(q\right)/I_{m}$ using the fitting function $F\left(q;R_{1},R_{2},\chi \right)=N_{min}{\left[\Gamma \left(q,R_{1},R_{2}\right)-2\chi \right]}^{-1}$ with the fitting parameters $R_{1}$, $R_{2}$ and χ for a given set of molecular parameters (Table 1), where $N_{min}$ is the minimum value of $\Gamma (q,R_{1},R_{2})-2\chi$. The complete formulas for the fitting function with molecular parameters are documented in Appendix A.

**Figure 2:**
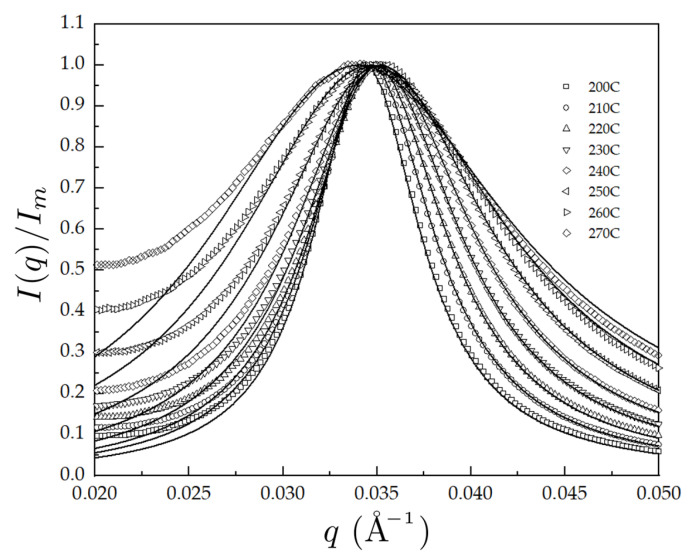
The normalized scattering profiles $I\left(q\right)/I_{m}$ fitted to $F(q;R_{1},R_{2},\chi )$ at various temperatures in the disordered state for PBA-*b*-PMMA.

Figure 2 shows the normalized scattering profiles $I\left(q\right)/I_{m}$ fitted to $F(q;R_{1},R_{2},\chi )$ at various temperatures in the disordered state, by which the temperature dependence χ(T) can be obtained assuming the linear relation $\chi =\chi_{S}+\chi_{H}/T$ in the mean-field regime where $\chi_{S}$ and $\chi_{H}$ are the temperature-independent constants associated with the entropic and enthalpic contribution to the overall χ[23,24,25,26]. It is worth commenting that although the peak fits well to the RPA model in the region *q* > 0.03 Å${}^{-1}$, the agreement in the low *q* region is poor, which is also observed in the similar previous works on the χ measurements using SAXS [27,28,29]. While this could be due to the slope in the SAXS data or background mismatch, there are also errors due to the polydispersity of the block copolymer and the conformational asymmetry between blocks [27]. As for the present work, since the corrections for polydispersity and asymmetry were taken into account in our RPA equations, it is likely that this error in the low q region is mainly due to the slope in the SAXS data. Nonetheless, the estimation of χ, which uses the inverse proportionality between χ and the width of scattering profile in the *q* region at 0.03 Å${}^{-1}$ < *q* < 0.04 Å${}^{-1}$, is unaffected because the peak width is independent of the slope in the SAXS.

**Figure 3:**
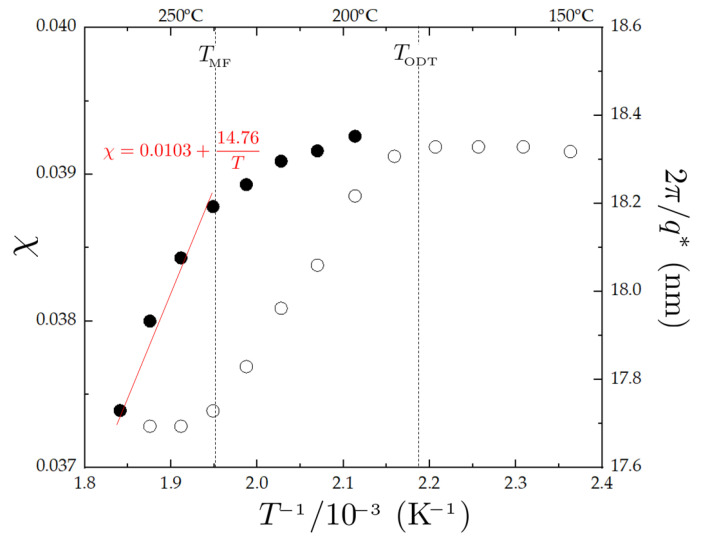
Temperature dependence of χ values and the characteristic length (2π/q*) for PBA-*b*-PMMA. The mean-field ($T>T_{\mathrm{MF}}$) and the ordered region ($T<T_{\mathrm{ODT}}$) are identified by two plateau regions in the $T^{-1}-2\pi /q^{*}$ plot. The red solid line represents the linear regression of ($T^{-1},\chi$) data in the mean region.

Figure 3 shows the temperature dependence of the estimated χ values and the characteristic length 2π/q*, where q* is the dominant wave vector in the scattering profile such that $I_{m}=I\left(q^{*}\right)$. The two plateau regions in the plot of $T^{-1}-2\pi /q^{*}$ also identify the mean-field ($T>T_{\mathrm{MF}}$) and the ordered region ($T<T_{\mathrm{ODT}}$), respectively. The resultant temperature dependence χ(T), which was obtained by the linear regression in the mean-field regime, was found to be

(1) $$\begin{array}{c} \chi =\left(0.0103\pm 0.0031\right)+\frac{\left(14.76\pm 1.64\right)}{T} \end{array}$$

where the thermodynamic temperature is used for *T*.

It is of interest to compare our estimation of χ(T) for PBA-*b*-PMMA to those for other diene-based block copolymer systems widely used for TPE, such as polystyrene-polybutadiene-polystyrene (SBS) or polystyrene-polyisoprene-polystyrene (SIS) triblock copolymers. Table 2 compares some of the available χ(T) measured for the polystyrene-*b*-polybutadiene diblock (PS-*b*-PB) and the polystyrene-*b*-polyisoprene diblock (PS-*b*-PI) to that of our system of PBA-*b*-PMMA.

As shown in Table 2, the temperature dependence of χ, which can be characterized by the enthalpic contribution $\chi_{H}$, is weaker for the PBA-*b*-PMMA block copolymer than that for diene-based block copolymers. For instance, χ values in the temperature range of 100–200 °C are 0.042–0.050 for PBA-*b*-PMMA, 0.032–0.046 or 0.032–0.048 for PS-*b*-PB, and 0.062–0.076 or 0.046–0.083 for PS-*b*-PI, which suggests less temperature-dependent conformational and morphological behavior for PBA-PMMA-based block copolymers than those for the conventional styrene-diene-based block copolymers.

## 4. Conclusions

In conclusion, we report the temperature dependence of the Flory–Huggins interaction parameter χ(T) between BA and MMA components by analyzing SAXS measurements fitted to RPA equations for a molten PBA-b-PMMA diblock at various temperatures. It was found from the χ estimation that the BA-MMA interaction is less susceptible to temperature than the interactions of styrene-diene-based block copolymers such as SIS and SBS triblock copolymers used commercially for TPE applications. Since the fraction of bridging conformation of triblock copolymer, a key for mechanically resilient TPE, is proportional to $\chi^{-1/9}$, this weaker temperature dependence of χ between BA and MMA provides a desirable option when designing terpolymers for developing thermally stable TPE.

## Figures and Tables

**Table 1:** Molecular parameters of PBA-*b*-PMMA used for fitting with the fitting function $F(q;R_{1},$$R_{2},\chi )=N_{min}{\left[\Gamma \left(q,R_{1},R_{2}\right)-2\chi \right]}^{-1}$ using Equations (A1)–(A3).

Volume Fraction of PBA Block ($f_{1}$)	Dispersity of PBA Block ($\lambda_{1}$)	Dispersity of PMMA Block ($\lambda_{2}$)	Total Number of unit Segments ($\bar{N}$)
0.553	1.084	1.579 ^1^	208.2 ^2^

^1^ Parameterized by λ − 1 = (λ_1_ − 1)$w_{1}^{2}$ + (λ_2_ − 1)$w_{2}^{2}$ with λ = 1.151, λ_1_ = 1.084, and *w*_1_ = 0.53 = 1 − *w*_2_, where $w_{\alpha }$ is the weight fraction of the component *α*. ^2^ Parameterized by $\bar{N}=\left(v_{1}N_{1}+v_{2}N_{2}\right)/$${\left(v_{1}v_{2}\right)}^{1/2}$ with *N*_1_ = 97.3, *N*_2_ = 110.1, *v*_1_ = 118.7 cm^3^/mol and *v*_2_ = 84.7 cm^3^/mol, where $N_{\alpha }$ and $v_{\alpha }$ are the number-averaged degree of polymerization and the molar volume of the component *α*, respectively.

**Table 2:** χ(T) reported for various diene-based block copolymers compared to that of PBA-*b*-PMMA.

System	χ(T)	Ref
PS-*b*-PB ^1^	−0.021+25/T	[30]
PS-*b*-PB ^2^	−0.027+28/T	[31]
PS-*b*-PI ^3^	−0.0090+25/T	[30]
PS-*b*-PI ^4^	−0.0937+66/T	[32]
PBA-*b*-PMMA	0.0103+14.76/T	

^1^ 1,2 and 1,4 diene microstructures are 95% and 5%, respectively. ^2^ Diene microstructures are unspecified. ^3^ 1,4 and 3,4 diene microstructures are 93% and 7%, respectively. ^4^ 1,2, 1,4 and 3,4 diene microstructures are 38%, 3% and 59%, respectively.

## Data Availability

The data presented in this study are available on request from the corresponding author.

## Appendix A

Here, we briefly summarize the fitting function $F\left(q;R_{1},R_{2},\chi \right)=N_{min}{\left[\Gamma \left(q,R_{1},R_{2}\right)-2\chi \right]}^{-1}$, which was used for the fitting SAXS data, $I\left(q\right)/I_{m}$, to extract χ of PBA-*b*-PMMA. The fitting function is based on the second-order vertex function Γ(q) related to the single chain correlation functions between the component α and β in the ideal binary system, $g_{\alpha \beta }$ (α,β = 1 (PBA), 2 (PMMA)) in the RPA equation [33]:

(A1) $$\begin{array}{c} \Gamma \left(q\right)=\frac{\sum_{\alpha \beta }g_{\alpha \beta }\left(q\right)}{|g_{\alpha \beta }|} \end{array}$$

The single chain correlation functions between the same species ($g_{11}$, $g_{22}$) and between the unlike species ($g_{12}$) for ideal diblock copolymer are given as

(A2) $$\begin{array}{c} g_{11}\left(q\right)=\frac{2\bar{N}f_{1}^{2}}{x_{1}^{2}}\left[x_{1}-1+u\left(\lambda_{1},x_{1}\right)\right] \end{array}$$

(A3) $$\begin{array}{c} g_{12}\left(q\right)=g_{21}\left(q\right)\frac{2\bar{N}f_{1}f_{2}}{x_{1}x_{2}}\left[1-u(\lambda_{1},x_{1})\right]\left[1-u(\lambda_{2},x_{2})\right] \end{array}$$

and $g_{22}$ is obtained by switching the indices of species from 1 to 2 in Equation (A2). In Equations (A2) and (A3), $\bar{N}$ is the number of segments (or the number of reference volumes) in the diblock, $f_{\alpha }$ is the volume fraction of the component α, $x_{\alpha }=q^{2}R_{\alpha }^{2}$ is the square of the scaled wave vector where $R_{\alpha }$ is the root-mean-square radius of gyration of the component α, and $u(\lambda_{\alpha },x_{\alpha })$ is the function for taking into account the dispersity $\lambda_{\alpha }$ of the component α, which is given by [27,34]

(A4) $$\begin{array}{c} u\left(\lambda_{\alpha },x_{\alpha }\right)={\left[x_{\alpha }\left(\lambda_{\alpha }-1\right)+1)\right]}^{-\frac{1}{\lambda_{\alpha }-1}} \end{array}$$

The total number of segments, $\bar{N}$, can be further expressed by $\bar{N}=\left(v_{1}N_{1}+v_{2}N_{2}\right)/$${\left(v_{1}v_{2}\right)}^{1/2}$, where $N_{\alpha }$ and $v_{\alpha }$ are the number-averaged degrees of polymerization and the molar volume of the component α, respectively, and given for the present PBA-*b*-PMMA system as $N_{1}$ = 97.3, $N_{2}$ = 110.1, $v_{1}$ = 118.7 cm${}^{3}$/mol and $v_{2}$ = 84.7 cm${}^{3}$/mol. Lastly, the dispersity of the second block component (2:PMMA) in the ATRP, $\lambda_{2}$, was obtained by using the rule for the sum of variances of the distributions: $\lambda -1=\left(\lambda_{1}-1\right)w_{1}^{2}+\left(\lambda_{2}-1\right)w_{2}^{2}$ [35]. Here, λ and $\lambda_{1}$ are the overall dispersities of the block copolymer, the dispersity of the first block component (1:PBA), and $w_{\alpha }$ is the weight fraction of the component α, respectively, which are given for the present PBA-*b*-PMMA system as λ = 1.151, $\lambda_{1}$ = 1.084, and $w_{1}$ = 0.53.
